# Novel variations in the adiponectin gene (*ADIPOQ*) may affect distribution of oligomeric complexes

**DOI:** 10.1186/2193-1801-1-66

**Published:** 2012-12-14

**Authors:** Leah C Kottyan, Jessica G Woo, Mehdi Keddache, Walter Banach, Nancy A Crimmins, Lawrence M Dolan, Lisa J Martin

**Affiliations:** 1 Cincinnati Children’s Hospital Medical Center, 3333 Burnet Avenue, MLC 4006, Cincinnati, OH 45229 USA; 2 University of Cincinnati School of Medicine, Cincinnati, OH USA

**Keywords:** Type 2 Diabetes, Insulin resistance, Extreme phenotypes, Non-synonymous, Obesity, Genetic

## Abstract

Adiponectin is an obesity related protein that mediates the risk of type 2 diabetes in obese individuals with its anti-inflammatory and insulin-sensitizing properties. To date, five functional variations have been identified in the adiponectin gene. However, these variations are rare, and fail to fully explain adiponectin variability, suggesting unidentified causal variations exist. Thus, our objective was to identify novel, potentially functional amino acid-changing variations in *ADIPOQ* exonic regions and relate them to oligomeric forms of adiponectin in serum. We sequenced *ADIPOQ* exons in 30 adolescents chosen from a school-based cohort based on serum adiponectin and insulin levels. Four coding region changes were identified: a methionine initiation skip (MIS), P32L, R55C, and Y111H, of which R55C and Y111H have been previously identified. Individuals with the novel variations and R55C had low levels of adiponectin and decreased adiponectin oligomerization compared to adolescents with similar body mass index and insulin levels. Further, bioinformatic analysis predicted putative functionality of these variations. In our study, Y111H was unrelated to total circulating adiponectin or adiponectin oligomerization. Given the disruption of adiponectin oligomerization in the individuals with MIS, P32L, and R55C coding changes, these variations may lead to increased metabolic disease risk and warrant further examination in larger cohorts.

## Introduction

Obesity is a major problem in the United States with over two thirds of adults and one third of adolescents classified as overweight or obese Ogden et al. (2012). While excess weight is a risk factor for type 2 diabetes, most obese individuals do not develop type 2 diabetes (T2D) (Boyle et al. 2010; Eckel et al. 2011; Writing Group for the Search for Diabetes in Youth Study Group et al. 2007). Adiponectin may help explain the increased T2D risk in some obese individuals. Adiponectin has anti-inflammatory and insulin-sensitizing properties, and low adiponectin levels precede development of insulin resistance and T2D (Hotta et al. 2001; Lindsay et al. 2002). Mechanistically, adiponectin facilitates insulin actions in peripheral tissues by activating AMP kinase and p38 MAPK (Combs et al. 2001; Yamauchi et al. 2002). However, adiponectin’s effects on insulin sensitivity are more clinically relevant in obese individuals. In lean individuals Martin et al. (2005) and mice Maeda et al. (2002), adiponectin concentrations are not associated with insulin sensitivity; yet, in the context of obesity adiponectin concentrations show strong association with insulin levels (Maeda et al. 2002; Martin et al. 2005).

Adiponectin is coded by *ADIPOQ* (NCBI GeneID 9370) located at 3q27. *ADIPOQ* single nucleotide variations (SNVs) have been associated with T2D (p < 10^-2^), insulin resistance (p < 10^-2^), and serum adiponectin (p < 10^-8^) (Vasseur et al. 2002; Filippi et al. 2004; Hivert et al. 2008; Mackevics et al. 2006; Mousavinasab et al. 2006). Adiponectin multimerizes resulting in four circulating oligomeric forms: trimers (LMW), hexamers (MMW), high molecular weight (HMW), and very high molecular weight (VHMW) complexes Tsao et al. (2002). HMW and VHMW complexes are most active biologically Pajvani et al. (2004). To date, five causal SNVs have been described (R55H, G84R, G90S, R112C, and R131H). These SNVs are associated with hypoadiponectinemia, impaired adiponectin multimerization, and T2D (Waki et al. 2003; Takahashi et al. 2000; Jungtrakoon et al. 2011). However, these SNVs are rare and fail to fully explain adiponectin variability, suggesting unidentified causal SNVs exist.

Thus, our objective is to identify novel and known *ADIPOQ* coding changes and relate them to oligomeric forms of adiponectin. By sequencing *ADIPOQ* in adolescents with extreme serum adiponectin levels (high or low), we enriched our study group for non-synonymous *ADIPOQ* SNVs. In this article, we present *ADIPOQ* coding region variations and their effect on adiponectin oligomerization. While the impact of these SNVs may be masked in lean individuals, once validated in mechanistic studies, these mutations could be included in a metric used by clinicians to council at-risk children on their increased risk for insulin resistance before they become overweight and insulin-insensitive.

## Results

**Figure 1:**
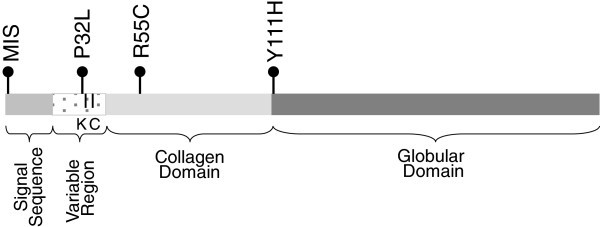
***ADIPOQ*gene structure and location of identified coding changes.** MIS, P32L, R55C, and Y111H. K is the position of a hydroxylated and glycosylated lysine. C is the position of a cysteine forming disulfide bonds.

Four non-synonymous SNVs were identified: a methionine initiation skip (c.130 G > A abbreviated as MIS), P32L, R55C, and Y111H (Figure 1). Each SNV was present as a heterozygote and found in a single individual. Three of the 4 SNVs (MIS, P32L, and R55C) were identified among the 10 (30% success rate) individuals with the lowest 1 percent of adiponectin in our cohort very low serum adiponectin. In the NHLBI’s Exome Project, only 21 non-synonymous *ADIPOQ* changes were identified in 5138 individuals: a 0.4 percent success rate. Selecting individuals with the lowest adiponectin levels to sequence led to a statistically significant enrichment for non-synonymous changes as compared to the NHLBI sequencing project (Fisher’s exact test p value < 10^-5^).

**Figure 2:**
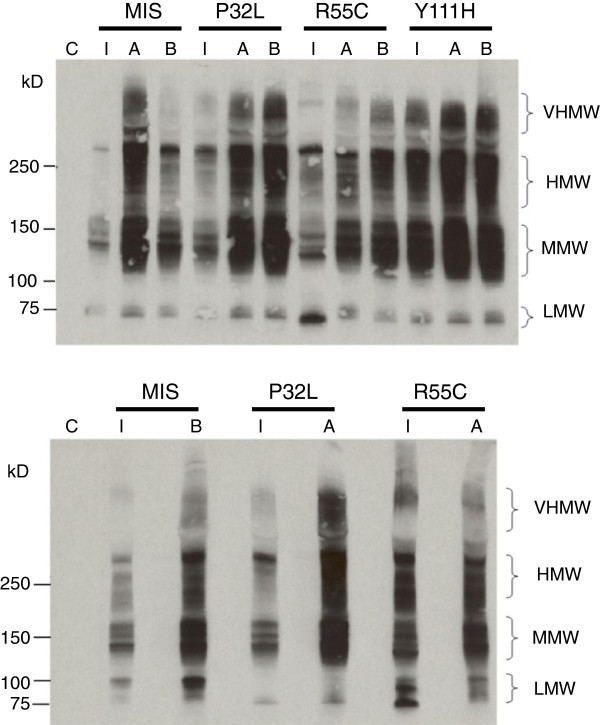
**Adiponectin oligomerization in serum using Western blotting on non-reducing gels.** C, negative control of loading buffer only. V, individual with variation. M, matched control. MM, mismatched control. Due to the low concentrations of adiponectin in some samples, gels were run using two different concentrations (Panel A: 2uL, Panel B: 4 uL).

Individuals with coding SNVs exhibited phenotypic heterogeneity with respect to BMI and insulin (Table 1), but glucose values were clinically normal and thus not presented. Interestingly, individuals with adiponectin SNVs and low adiponectin values had impaired oligomerization as evidenced by Western blots (Figure 2) and oligomeric ELISAs (Table 2) even when accounting for variability in BMI and insulin.

**Table 1:** **Demographic Characteristics of Individuals with Variations (SNV) and Matched Controls**

SNV	Subject^a^	Age	Sex	Race	Puberty	BMI (kg/m^2^)	BMI Z	Insulin (pmol/l)
MIS	Index	14.7	M	W	Post	21.7	0.67	25.6
	Match – A	14.9	M	W	Post	21.4	0.54	44.0
	Match - B	15.1	M	W	Post	22.3	0.74	133.7
P32L	Index	12.8	F	W	Peri	23.1	1.19	330.9*
	Match - A	12.6	F	W	Peri	22.9	1.19	202.7*
	Match - B	13.0	F	W	Peri	22.3	0.99	112.9
R55C	Index	13.4	M	B	Peri	33.3	2.38	350.2*
	Match - A	13.7	M	B	Peri	35.9	2.51	330.6*
	Match - B	13.7	M	B	Peri	29.1	2.04	84.9
Y111H	Index	11.8	F	W	Peri	18.3	0.14	74.0
	Match - A	12.0	F	W	Peri	18.5	0.17	77.8
	Match - B	12.1	F	W	Peri	18.7	0.20	25.3
5th - 95th Percentiles^b^		10.8 – 17.9				16.5 – 34.6	−1.02 – 2.35	35.0 – 348.2

* Insulin resistant.

^a^ “Index” refers to the individual carrying the variation noted. Both Match - A and Match - B subjects were sequenced to verify that they did not harbor non synonymous SNVs in *ADIPOQ*. Match- A refers to the individual selected to match the Index individual by age, sex, race, puberty status, BMI and insulin levels. Match – B refers to the individual selected to match the Index by age, sex, race, puberty status, and BMI, but to be as divergent on insulin levels. ^b^ Percentiles derived from the larger cohort of 2501 children.

**Table 2:** **Quantification of serum adiponectin and oligomerization**

SNV	Subject^a^	RIA	ELISA					
		Total (μg/ml)	Total (ug/ml)	HMW + MMW (ug/ml)	HMW (ug/ml)	MMW (ug/ml)	LMW (ug/ml)	% HMW
MIS	Index	0.6	1.1	0.4	0.03	0.37	0.7	0.03
	Match – A	11.6	7.5	5.5	2.9	2.6	2	0.39
	Match - B	3.1	2.6	1.7	0.5	1.2	0.9	0.19
P32L	Index	2.3	2.2	1.3	0.6	0.7	0.9	0.27
	Match - A	12.3	6.4	4.9	2.6	2.3	1.5	0.41
	Match - B	12.7	7	5.3	3.4	1.9	1.7	0.49
R55C	Index	1.2	4.3	0.4	0	0.4	3.9	0.00
	Match - A	4.9	3.4	2	0.7	1.3	1.4	0.21
	Match - B	11.1	4.8	3.1	1.9	1.2	1.7	0.40
Y111H	Index	13.6	6	5.1	2.6	2.5	0.9	0.43
	Match - A	13.2	8.7	7.3	4.3	3	1.4	0.49
	Match - B	12.6	8.3	6.3	3.7	2.6	2	0.45

Total adiponectin (Total APN), high molecular weight adiponectin (HMW APN), medium molecular weight adiponectin (MMW APN), and low molecular weight adiponectin (LMW APN) were measured by ELISA for each SNV in the subject with the variant and a matched and mismatched control. %HMW: the percentage of adiponectin present in the high molecular weight form.

^a^ “Index” refers to the individual carrying the variation noted. Both Match A and Match B subjects were sequenced to verify that they did not harbor non synonymous SNVs in *ADIPOQ*. Match- A refers to the individual selected to match the Index individual by age, sex, race, puberty status, BMI and insulin levels. Match – B refers to the individual selected to match the Index by age, sex, race, puberty status, and BMI, but to be as divergent on insulin levels. ^b^ Percentiles derived from the larger cohort of 2501 children.

### Identification of novel adiponectin SNV: MIS

Our participant with MIS had the lowest adiponectin in the cohort; however, this individual exhibited normal adiposity (CDC BMI percentile < 85) and insulin. Based on Western blot, there was little HMW and no very HMW (VHMW) adiponectin; ELISA confirmed these findings with only 2.7% of adiponectin coming from HMW. SIFT and PolyPhen-2 suggests that this change could be tolerated and benign, respectively, at a protein level. However, this variation is predicted to eliminate the first 39 amino acids (p.Met1_Trp39del, http://www.expasy.ch.spdbv) which would eliminate the signaling peptide component of the protein (http://www.uniprot.org/uniprot/Q15848).

### Identification of novel adiponectin SNV: P32L

The individual with P32L exhibited low (below the 1st percentile of the cohort) adiponectin and was hyperinsulinemic and overweight (BMI between CDC 85th and 95th percentiles). Based on the Western Blot, this individual also had very low VHMW adiponectin, in contrast to both of her insulin resistant match and insulin sensitive mismatch controls, but surprisingly similar to the individual with MIS (Figure 2). Based on the ELISA, this individual had substantially lower HMW adiponectin oligomers (27%) as compared to her match and mismatch controls (41% and 49%, respectively). Because the SNV falls within the hypervariable region of *ADIPOQ* (Figure 1), the SIFT prediction that this amino acid is not highly conserved is expected (Table 3). In agreement with our biological findings, the PolyPhen-2 score predicts that this change is possibly damaging (Table 3).

**Table 3:** **In silico assessment of rare*ADIPOQ*variations (SNV)**

SNV	POS	rs number	SIFT				PolyPhen-2			
			Prediction	Score	Median Information Content	# Seqs	HdivPred	HDivProb	HvarPred	HVarProb
MIS	186570850	--	-	-	-	-	Benign	0.141	Benign	0.028
P32L	186570942	--	Tolerated*	0.11*	2.32	99	Possibly Damaging	0.956	Possibly Damaging	0.478
R55C	186571010	rs138227502	Damaging	0.05	2.22	163	Probably Damaging	1	Probably Damaging	1
Y111H	186572089	rs17366743	Tolerated	0.55	2.15	185	Benign	0.006	Benign	0.012
R112C	186572092	rs79645624	Damaging	0.05	2.15	185	Probably Damaging	1	Probably Damaging	0.947

SIFT and PolyPhen-2 were utilized to predict the possible impact of amino acid substitutions on the structure and function of *ADIPOQ* (ESNP00000389814, NP_001171271.1). Position (POS) is based on NCBI Build 37. R112C is the mutation known to cause Adiponectin deficiency Takahashi et al. (2000). SIFT: The SIFT Score ranges from 0 to 1. The amino acid substitution is predicted damaging if the score is < = 0.05, and tolerated if the score is >0.05. The Median Information Content gives the diversity of the sequences used for prediction. The # Seqs gives the number of sequences that have an amino acid that the position of prediction. There were insufficient sequences to predict the effect of MIS using the SIFT algorithm. *Given the fact that P32L falls in a evolutionary hyper variable region, the tolerated prediction is expected. PolyPhen-2: the naive Bayes posterior probability that a given mutation is damaging and reports estimates of false positive (the chance that the mutation is classified as damaging when it is in fact non-damaging) and true positive (the chance that the mutation is classified as damaging when it is indeed damaging). HdivPred and HVarPred are predictions based upon distinct learning sets.

### Identification of adiponectin SNV R55C

Our participant with R55C had low adiponectin and was insulin resistant and was obese (BMI ≥ CDC 95th percentile). This SNV was recently reported as part of the NHLBI Exome Project, but no phenotypic information was provided. Western blot shows a lack of VHMW and little HMW adiponectin, with a significant LMW band at 2μL (Figure 2). At 4μL, a double LMW band is observed, perhaps indicating a dimer, which was not seen in other samples. From the ELISA, HMW adiponectin is not detectable. Strikingly, the SIFT/Polyphen-2 scores for this mutation predict a damaging/probably damaging effect equal in magnitude to the causal R112C mutation (Table 3).

### Identification of adiponectin SNV Y111H

The individual with this previously reported SNV had Type 1 Diabetes and total adiponectin levels above our cohort mean. This individual was lean and showed no differences in circulating oligomeric structures relative to either her match or mismatch controls. SIFT and PolyPhen-2 scores for this change predict that Y111H is tolerated and benign (Table 3).

## Discussion

As adiponectin is associated with obesity-related risk of T2D and is strongly determined by genetic factors, our objective was to identify novel and known *ADIPOQ* coding changes and relate their relationship to oligomeric forms of adiponectin. Using an extreme discordant phenotyping approach, four non-synonymous heterozygous *ADIPOQ* changes were identified, two of which were novel. By sequencing individuals with extremely low serum adiponectin levels we enriched the sample for non-synonymous SNVs as compared to a sample sequenced without respect to adiponectin levels. Three of the four SNVs exhibited impaired oligomerization consistent with bioinformatic predicted functional effect. Once biologically validated, these variations could be used to identify individuals at-risk of obesity related insulin resistance and T2D.

In the three index individuals with non-synonymous SNVs and low serum adiponectin level, BMI and insulin levels exhibited variability; 2 of the 3 individuals were obese and hyperinsulinemic. Yet, the oligomeric patterns in these three index individuals were consistent with reduced HMW adiponectin compared to individuals with similar insulin levels, suggesting a potential dominant negative effect. MIS and P32L have not been reported in dbSNP135, the Human Gene Mutation Database at the Institute of Medical Genetics in Cardiff, or the NHLBI Exome Project. P32L and R55C were both predicted to be potentially damaging by PolyPhen. For P32L, reduced functionality may be due to the proximity of the SNV to a conserved lysine (K33) and a cysteine (C36) known to form disulfide bonds (Figure 1) Waki et al. (2003). R55C was initially reported as part of NHLBI’s Exome Sequence Project (rs138227502, frequency 0.1%), but no phenotypic information was provided. R55C introduces a novel cysteine 19 amino acids downstream of C36, potentially affecting disulfide bond formation. Interestingly, in Thai T2D patients, a similar SNV, R55H resulted in substantially reduced HMW formation as compared to wild type Jungtrakoon et al. (2011). On the other hand, neither SIFT nor PolyPhen predicted a damaging effect of MIS. However, it is important to recognize that PolyPhen does not account for the amine portion of *ADIPOQ* where this variation occurs. Indeed, adiponectin missing the N-terminal region, similar to MIS, has reduced ability to inhibit collagen-induced platelet aggregation and diet induced hepatic steatosis Ujiie et al. (2006). Further, the presence of a normal metabolic profile in our subject is consistent with the adiponectin knockout mouse, whose insulin and glucose remained normal until placed on a high-fat diet Maeda et al. (2002).

Y111H has been reported (rs17366743) (Vasseur et al. 2002; Waki et al. 2003; Kretowski et al. 2005), with a minor allele frequency of 5.8%. While previous reports suggest adiponectin levels were reduced with this SNV (Vasseur et al. 2002; Kretowski et al. 2005), the individual with this change had adiponectin levels above the mean for our cohort consistent with higher adiponectin levels seen in individuals with Type I diabetes (Morales et al. 2004; Frystyk et al. 2005). Further, this SNV did not appear to alter adiponectin oligomeric distribution.

In this paper, we report identification of three putatively functional heterozygous *ADIPOQ* SNVs after sequencing ten individuals with the lowest adiponectin in our cohort. Given our high rate of success in identifying coding SNVs, sequencing candidate genes and an extreme phenotype approach may be an excellent mechanism to complement current exome sequencing approaches. Importantly, our approach identified rare heterozygous non-synonymous SNVs; evaluation of these heterozygous non-synonymous changes can be challenging in exome sequencing data as previous studies have demonstrated that thousands of non-synonymous SNVs may be present in any one individual Wheeler et al. (2008). Given the large number of non-synonymous heterozygous changes, the likelihood of any one heterozygous SNV being overlooked might be quite substantial. Indeed, while R55C was identified in the NHLBI exome sequencing project, there are no studies examining its effect. This could be a problem for adiponectin as all functional SNVs are heterozygous (Jungtrakoon et al. 2011; Kondo et al. 2002; Vasseur et al. 2002).

As adiponectin plays an important role in mediating obesity related insulin resistance (Martin et al. 2005; Maeda et al. 2002) especially the high molecular weight forms Pajvani et al. (2004), identification of functional adiponectin variations could have substantial clinical implications. Specifically, if these variations cause hypoadiponectinemia, then individuals with these changes may need to be counseled to more aggressively maintain a healthy weight and low carbohydrate diet, as these individuals will be at highest risk for hyperinsulinemia and T2D. However, one limitation of this study is the small sample size examined, so it is not possible to ascertain from this study whether these variations may occur in other patients and lead to the same findings with regard to oligomerization. It is therefore important to validate these findings biologically and clinically first. For example, examining adiponectin serum levels and oligomerization in the 21 individuals from NHLBI’s Exome Sequencing Project with an R55C variation could provide additional support that this variation is likely functional.

It is important to recognize that while this investigation focused on coding changes that potentially affect adiponectin levels and oligomerization, other variations exist in the 5’ regions of the gene that are associated with adiponectin gene expression or serum levels. In particular, we and others have reported that variations between 10 kb and 12 kb upstream of the *ADIPOQ* transcription start site may alter adiponectin levels (Woo et al. 2006; Heid et al. 2010; Gupta et al. 2012), and proximal promoter polymorphisms have been implicated as well Laumen et al. (2009). Because individuals were selected for sequencing and Western blotting based only on phenotype, many also harbored various promoter region, synonymous and intronic variations previously genotyped in this population Woo et al. (2006). However, in a randomly selected set of 54 individuals from this cohort (data not shown), only one individual had HWM as low as our individual with P32L and none as low as our individuals with R55C and MIS. Thus, it is unlikely that these other variations are responsible for the very low HWM adiponectin present in our individuals with structural changes.

## Conclusions

In summary, we identified two novel non-synonymous *ADIPOQ* variations using an extreme phenotype sequencing approach. Individuals with these novel variations had low adiponectin and exhibited reduced HMW structures compared to individuals without these variations. Although each variation is present in the heterozygous state, the effects may be dominant negative. This study highlights the utility of sequencing individuals with extreme phenotypic values to discover novel coding changes. These variations may provide important clues to the relationships among *ADIPOQ* genetic variations, adiponectin oligomeric distribution, and total circulating adiponectin levels. Indeed, future studies might well demonstrate that rare potentially causal changes identified in this study contribute to the statistical association of more common variations with metabolic phenotypes in larger populations.

## Methods

### Human subjects protection

This study was approved by Cincinnati Children’s Hospital Medical Center Institutional Review Board. All participants or parents/guardians provided written informed consent. Subjects under 18 years of age provided assent.

### Population

**Figure 3:**
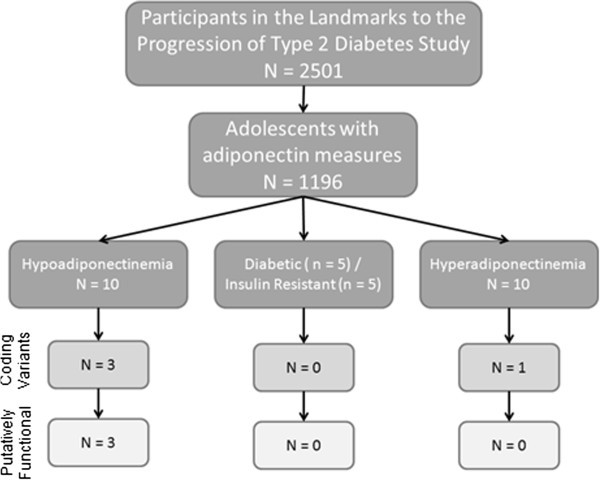
**Study design led to enrichment for subjects with missense changes.**

The overall study design is an extreme phenotype approach (Figure 3). Briefly, 30 participants with abnormal adiponectin levels or specific phenotypes were selected for *ADIPOQ* sequencing; 10 with the lowest adiponectin levels (range 0.6 – 2.5 μg/mL), 10 with the highest adiponectin levels (range 19.9 – 29.15 μg/mL), 5 diabetic (4 T2D, 1 T1D) and 5 with insulin resistance (range 127 – 1327 pmol/L insulin). Of these individuals 48 percent were male, the mean age was 14.4 years, and there were equal numbers of blacks and whites.

All 30 participants were selected from a larger cohort of 1196 black and white students who had adiponectin levels assayed Martin et al. (2005). The 1196 participants were, in turn, randomly selected from a study of 2501 students from the Princeton City School District (Cincinnati, OH), who were enrolled from grades five through 12 to participate in a school-based study of carbohydrate metabolism Dolan et al. (2005). Height and weight were measured and blood samples were collected.

### Laboratory assays

Fasting serum insulin, glucose, and adiponectin were assayed as described previously (Martin et al. 2005; Dolan et al. 2005). Oligomeric adiponectin assays (Western Blots and ELISAs) were performed in 12 individuals. For Western Blots, SDS-PAGE was performed according to Laemmli’s method Laemmli (1970). Sample buffer for non-reducing, non-heat-denaturing conditions was 2% SDS, 50 mM Tris–HCl pH 6.8, and 10% glycerol. Samples were incubated overnight in sample buffer at 4°C and separated using 4-12% SDS-PAGE gradient gels (Jules, Inc., Milford, CT) at 80 V constant voltage. Proteins separated by SDS-PAGE were transferred to nitrocellulose membranes and blocked with Tris-buffered saline with 0.5% Tween containing 3% skim milk and incubated with a polyclonal antibody specific for human *ADIPOQ* (0.2 ug/ml; R&D Systems, Minneapolis, MN) overnight at room temperature. After rigorous washing, membranes were incubated with HRP-conjugated polyclonal donkey-anti-goat IgG (1:5000; Chemicon, Temecula, CA) for 1 hour at room temperature. Bands were detected using chemiluminescent detection reagent (Pierce, Rockford, IL) and exposure to x-ray film (Amersham, UK). LMW, MMW and HMW bands were classified as ~75kD, 135–150 kD and ~300 kD, respectively, consistent with previous reports (Pajvani et al. 2004; Waki et al. 2003); an observed higher kD band was classified as very high molecular weight (VHMW). To further explore oligomeric profiles, we used ALPCO HMW and Total Adiponectin ELISA per manufacturer’s instructions (ALPCO, Salem, NH) Ebinuma et al. (2006).

### Sequencing

DNA was extracted from frozen buffy coat using the Magnesil system (Promega Corporation, Madison, WI) automated on a KingFisher96 magnetic bead manipulator (Thermo Electron, Waltham, MA). Primers to amplify the *ADIPOQ* coding region (exon 2 and the 5^′^ end of exon 3, Table 4) were designed using ExonPrimer (http://ihg.gsf.de/ihg/ExonPrimer.html). The 1,954 bp fragment was amplified from 50 ng of genomic DNA using AmpliTaq Gold PCR system (Applied Biosystems, Foster City, CA) modified from standard conditions by increasing annealing temperature to 65°C and extension time to 3 minutes. PCR primers and nucleotides were enzymatically removed from the amplicon before direct sequencing using ExoSAP-IT (GE Healthcare, Fairfield, CT). A total of 4 sequencing reactions were performed to obtain bidirectional-sequencing of the entire coding region (primers in Table 4) using BigDye v3.1 Terminator kit on a 3730xl DNA Analyzer (Applied Biosystems). SNV analysis was performed using Mutation Surveyor v2.61 software (SoftGenetics, State College, PA).

**Table 4:** **ADIPOQ amplification and sequencing primers**

Primer	Sequence	Used for PCR	Used for Sequencing
*ADIPOQ*-F	GAGATGGACGGAGTCCTTTGTAGG	X	X
*ADIPOQ*-R	CTGGTCATGTTTGTGAAGCTCCC	X	X
*ADIPOQ*-1	ACACTCATCCTTGGAAGACCAACC		X
*ADIPOQ*-2	CCACAGGGATGGTAATTTAGGCTG		X

Individuals with non-synonymous coding changes were matched by age (± 6 months), puberty stage, sex, and race to individuals from the larger cohort. One control (Match-A) was chosen with similar BMI Z-score and insulin, given demographic matching. A second control (Match-B) was chosen, matching on demographic variables and BMI Z-score, but differing on insulin. Matched controls were sequenced to ensure they did not harbor *ADIPOQ* coding changes.

### Statistical analysis

BMI Z-score was calculated from CDC growth charts. Insulin resistance was defined as insulin levels >95%ile of lean individuals in the overall 2501-student cohort with the same race, sex and puberty stage Dolan et al. (2005). To determine whether individuals with *ADIPOQ* coding changes exhibited extreme phenotypes, we calculated the 5th and 95th percentiles for age, adiponectin, insulin, glucose, and BMI Z-score, using our cohort of 1196 students Martin et al. (2005). To compare the frequency of detecting non-synonymous SNVs to a public database (NHLBI Exome Sequencing Project), we used Fisher Exact Test.

### In silico investigation of non-synonymous SNVs

Two online bioinformatics tools, SIFT (http://sift.jcvi.org/) and PolyPhen2 (http://genetics.bwh.harvard.edu/pph2/), were used to predict the possible impact of amino acid substitutions on the structure and function of *ADIPOQ* using physical and comparative considerations. SIFT examines the evolutionary conservation of variations while PolyPhen2 predicts putative damaging effects of non-synonymous SNVs. SIFT predicts a damaging effect if the scaled probability score (SIFT score) is less than 0.05; otherwise, the algorithm predicts that the SNV will be tolerated (Adzhubei et al. 2010; Ng and Henikoff 2003; Kumar et al. 2009). PolyPhen-2 uses eight sequence-based and three structure-based predictive features to calculate the naïve Bayes posterior probability that a given mutation will be damaging and qualitatively predicts that it will be benign, possibly damaging, or probably damaging (Zou et al. 2011; Flanagan et al. 2010).

## Abbreviations

MIS: Methionine initiation skip
T2D: Type 2 Diabetes
SNV: Single nucleotide variation
LMW: Trimer
MMW: Hexamer
HMW: High Molecular Weight
VHMW: Very High Molecular Weight
BMI: Body Mass Index
CDC: Centers for Disease Control.
